# Healthy Ageing in Place: Enablers and Barriers from the Perspective of the Elderly. A Qualitative Study

**DOI:** 10.3390/ijerph17186451

**Published:** 2020-09-04

**Authors:** Cristina Bosch-Farré, Maria Carmen Malagón-Aguilera, David Ballester-Ferrando, Carme Bertran-Noguer, Anna Bonmatí-Tomàs, Sandra Gelabert-Vilella, Dolors Juvinyà-Canal

**Affiliations:** 1 Health and Healthcare Research Group, Department of Nursing, University of Girona, 17003 Girona, Spain; cristina.bosch@udg.edu (C.B.-F.); david.ballester@udg.edu (D.B.-F.); carme.bertran@udg.edu (C.B.-N.); anna.bonmati@udg.edu (A.B.-T.); dolors.juvinya@udg.edu (D.J.-C.); 2 Department of Nursing, University of Girona, 17003 Girona, Spain; sandra.gelabert@udg.edu

**Keywords:** healthy ageing, ageing in place, health promotion, elderly perspective

## Abstract

Background: Most elderly people wish to grow old at their own homes. The sociodemographic characteristics; home and neighbourhood conditions; and the social services support and networks are determinants in the possibility of “ageing in place”. The present study aimed to explore the ageing in place phenomenon, as well as the enablers and barriers that interact in a healthy ageing from the perspective of the elderly connected to local entities. Methods: A generic qualitative design was proposed in the Health Region of Girona in Catalonia (Spain). Seventy-one elderly people were purposefully selected. Six focus groups were conducted, and data were thematically analysed. Results: Three key themes were generated: (1) Participants experienced ageing differently. The physical and mental health, the family environment and financial stability were key elements for life quality. (2) The perception of the elderly’s role in the community depended on their age, health status and attitude towards life. (3) The participants identified several enablers and barriers to healthy ageing in place. Conclusions: The promotion of older people’s autonomy and wellbeing, together with the creation of an active network of health and social services, may improve the possibility for elderly to age at home and avoid or delay institutionalisation.

## 1. Introduction

Population ageing represents a global public health challenge. According to the United Nations, by 2050, one in every four persons in Europe and North America could be aged 65 years or over [1]. Europe is the continent with the oldest population and with the highest old-age dependency ratio [2]. Currently, 18.2% of the Europeans are older than 65 years old, and by 2050 this percentage is expected to increase to 28.1% [3]. Catalonia is among the European regions with the highest percentage of most elderly (≥85) among the 65 years-and-over population [4]. According to the World Health Organization (WHO), the extent of the opportunities that arise from increasing longevity will be heavily dependent on one key factor—The health of these older populations. Most healthcare problems in the elderly are related to chronic disorders, many of which can be prevented or delayed with health promoting behaviours [5].

The WHO and other international organisations declare Healthy Ageing (HA) as a priority [5,6]. The WHO defines HA as the process of promoting and maintaining the functional ability to enable well-being in older age [5]. This functional ability is determined by the individual’s intrinsic capacity (physic, mental and psychosocial), the environment (including physical, social and policy environment) and their interaction [5]. To achieve the goals of the Global Strategy and Action Plan on Ageing and Health, the WHO urges entities, not so much to increase longevity per se, but rather to focus on the quality of the extra years, encouraging older people’s activity, autonomy and integration in the society [7]. Specifically, one of the strategic objectives of the Global Strategy and Action Plan on Ageing and Health consists in improving the measurement, monitoring and research on HA [7]. For all these reasons, there is the need to identify the conditions in which people age and how these conditions influence their health, taking into account that the functional level of people is not only determined by their physical abilities, but also by the interaction with their environment throughout their lives [8].

The home is the main element of life quality in older people and a crucial tool to integrate ageing in society [9]. Over 80% of older people want to age at home [10,11,12,13]. In Spain, nine out of ten people show this preference, which is higher in men and for people under 80 years old [14]. “Ageing in place” refers to the ability to live in one’s own home and community safely, independently and comfortably, regardless of age, income or ability level [15]. Promoting HA, together with delaying the institutionalisation up to when it becomes strictly necessary [16], enables older people to age integrated in their communities. This strategy for ensuring health in older age is crucial to achieve sustainable development [17,18,19]. The main predictors of institutionalisation of older people described are the dependency level, the willingness to live in a residence, low support network and a diagnosis of dementia [20]. The older people who prefer to live in institutions have poorer health conditions and considerable disabilities, although better economic conditions to afford a place in a private residence [13].

The critical factors that contribute to ageing in place with quality are: habitat and safe environment, sufficient income to sustain life, support from family and friends and access to primary healthcare [21]. In this sense, it can be considered that ageing at home is a multidimensional concept that requires, among other aspects, to have adequate housing, a social network, specific sociodemographic characteristics and the provision of services and assistance. In a longitudinal study conducted in Australia, it was concluded that having socioeconomic resources, owning the house, being satisfied with the neighbourhood and making adaptations to the house are positively associated with ageing at home [18]. The support of the social and familiar environment is also a relevant element that can influence the ageing in place [22]. Furthermore, it has been observed that living at home is a relevant component of social connexion [23]. In this line, being less involved in social activities increases the likelihood of older people being moved from their homes [24]. Community services and a primary health system integrated and accessible to meet the needs of older people, have been stated to be essential for a successful home ageing [25].

Ageing in place is a key element of the quality of life of older people [23]. According to the point of view of older people, eight elements are crucial for ageing in place: health, information, practical assistance, financial conditions, activity (physical and mental), company (family, friends, neighbours and pets), transport and safety [25]. Personal characteristics that have been identified as contributing to the ability of ageing in place are resilience, adaptability and independence [25]. Ageing in place is related to a sense of identity both through independence and autonomy and through relationships and solidarity roles in the places people live [26]. However, there is little evidence about the factors that most influence on an ageing in place in quality, centred in the perception of the older people. Most studies are focused on one of the dimensions of health (physical, psychological or social), and there is little literature that addresses ageing from a multidimensional, salutogenic and person-centred perspective, through a qualitative approach. The present study was based on the hypothesis that the identification of the factors that impact on ageing in place will improve health and wellbeing of older people and will provide information to develop better care. The aim of the present study was to explore the ageing in place phenomenon and the different factors (enablers and barriers) that interact in HA from the point of view of older people connected to local entities of social support. Research on the ageing experience in a community environment will contribute to a better understanding of the ageing in place phenomenon. This, in turn, will allow for targeted policies and interventions aimed to HA promotion in the living environment, and thus better address the challenge of our ageing society.

## 2. Materials and Methods

### 2.1. Design

A generic qualitative design [27,28] with a constructivist naturalistic approach was proposed to analyse the interpretations attributed to the experience of ageing in place. The qualitative methodology offers a research tool that enables the understanding of the complexity of a phenomenon from the different points of view of the social actors involved in such phenomenon [29]. The constructivism considers reality to be an interpretation of the world [30]. There are as many realities as interpretations of such reality, and knowledge is created through the common characteristics of all the possible interpretations [30]. The naturalistic approach refers to the attempt to understand the phenomenon by the interpretation of the different subjective perceptions of the participants in the real context where the phenomenon takes place [31].

The present study proposed a generic qualitative design for the methodology. Such design, as studied by Caelli et al. [27], is useful in research that, in opposition to the phenomenology, fundamental theory and ethnography, is not within the limits of a unique established methodology. Such methodology enables the researcher to modify and adapt the study structure, resulting in a qualitative study design adjusted to the particular needs of the research conducted.

### 2.2. Study Area

The study area covered the Health Region of Girona in Catalonia (Spain) (see Appendix A) which comprised 218 municipalities and a population of 838,103 inhabitants in 2017. The life expectancy at birth was 82.95 years (80.14 for men and 85.81 for women); the ageing index, which refers to the ratio of the number of elderly persons of an age when they are generally economically inactive (aged 65 and over) to the number of young persons (from 0 to 14), was 106.87 (90.91 for men and 123.87 for women); and the percentage of the oldest-old (+85) among the 65 years-and-over population, was 16.7% (12.6% for men and 19.8% for women) [32].

### 2.3. Recruitment and Data Collection

This study was carried out between June 2017 and February 2018 using the focus group (FG) technique. Such technique is a suitable method to explore the experiences, opinions, wishes or concerns of individuals [33]. The FG technique enables, through the interaction within the respondents, the development of a discussion about a topic. The analysis of the information gathered enables to identify and to analyse the social meaning of the studied topic [33,34].

The sample size was selected based on the concept of information power and took into account the aim of the research, the sample specificity, the use of established theory, the quality of data and the analysis strategy [35]. Six FG were organised with elderly persons connected to local entities of social support in the municipalities of Girona (TA), Figueres (FI), Blanes (BL), Sant Feliu de Guíxols (SG), Olot (OL) and Sant Joan de les Abadesses (SJ). FG discussion participants were selected from local entities of social support for older people (such as community centres, older people’s associations and other similar institutions) in the Region of Girona (see Appendix B). The sessions took place in accessible community facilities chosen by the supervisors of the social support entities and scheduled at a suitable time for all participants. The meetings were held in neutral, quiet spaces, free of stimuli and mutually agreed by the participants and the moderator [33]. Each FG discussion had between 8 and 15 people, for a total of 71 participants [36], which ensured a sufficient sample size [33,34,37].

To recruit participants for the FG, purposive sampling was used [38]. The recruitment of participants was carried out through local entities of social support sufficiently large to ensure a sufficiently homogeneous experience of ageing in the community, as well as a sufficiently heterogeneous sample in terms of age, gender and rural or urban environment of the participants. Such heterogeneity of the sample units enabled the differences required for the discursive process and the identification of a larger range of factors related to the studied topic [33,34,37]. The inclusion criteria were: men and women aged 65 years and over who lived within their own community and who could speak Catalan or Spanish. The exclusion criteria were suffering cognitive impairment or not accepting the participation in the study.

**Figure 1:**
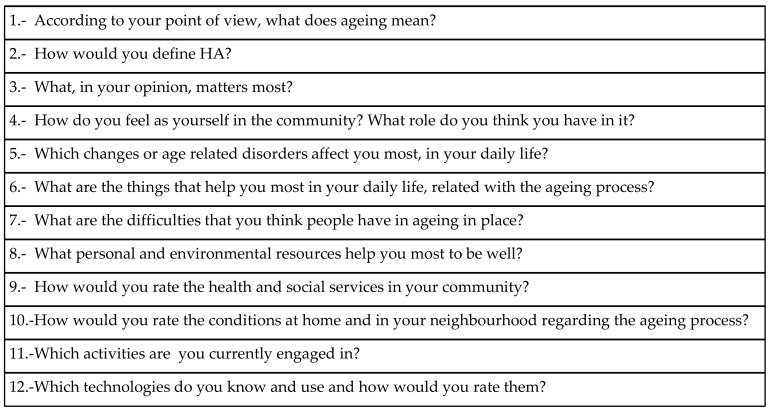
Semi-structured guide used in the focus groups.

During the FG sessions, a semi-structured guide with open questions related to the strategic objectives of the Global Strategy and Action Plan on Ageing and Health was followed [7]. Such semi-structured guide was developed based on the literature and on the indications suggested by Kvale [39]. The principal topics of the research (theoretical language) were converted into dynamic interview questions in the colloquial language of the participants. Several meetings within the researcher team were carried out to develop the 12 final questions of the semi-structured guide [36,39] (see Figure 1). The questions were in Catalan or Spanish depending on the context and the language of the majority of the participants. At the beginning of the FG sessions, information about age and gender was confidentially collected. Other demographic characteristics of the participants, such the rural or urban origin, were inferred from the locations of the FG meetings. Each FG session lasted 60–90 min and was conducted by a moderator and an observer [40]. Each session was digitally recorded, with the participant’s written consent. At the beginning of each session, a voice check was conducted, where the participants introduced themselves. Such procedure facilitates the subsequent voice recognition, which is one of the difficulties of the FG transcription [33].

### 2.4. Data Analysis

All interviews were transcribed. Inductive and deductive thematic analysis was employed to explore the data generated from the FG discussions. The six-step process proposed by Braun and Clarke [41] was followed. During a first phase of the analysis, the descriptive phase, data were organised to enable a description of the information obtained. This analysis was conducted holistically and line by line. As such, open codes were generated, which were arranged afterwards by criteria of similarity and thus defining the analysis categories [41,42,43]. During the second phase, the interpretative phase, the researchers sought patterns of meaning in the data, which significances were behind the themes and how these significances were aligned with the context of the research phenomenon [27,41].

To ensure a rigorous research, the present report was guided by the Consolidated Criteria for Reporting Qualitative Research (COREQ) [44]. Among other items, the researchers followed the criteria of reflexivity (constant critical attitude with the methodological decisions undertaken to safeguard the rigor and methodological congruence) [27] and positionality (the previous assumptions of the researchers regarding the research question were taken into account; additionally, the moderators of the FG considered their position in relation to the common characteristics of the group, the potential presence of power relations and the social desirability bias that can occur in the context of a group session) [33].

Ethical approval to conduct the study was received from the Ethics Committee of the Institut d’Investigació d’Atenció Primària Jordi Gol, (Idiap Jordi Gol) (CodeP17/104). All procedures were in line with the Declaration of Helsinki: all respondents willingly agreed to participate with a written consent; they received oral and written information about the study; and they were assured of the confidentiality of all information.

## 3. Results

A total of 71 people participated in the six FG. Participants ranged in age from 62 to 92 years, with a median of 75.39 years (6.94 SD). Regarding gender, 60.56% were women and 39.43% were men. Overall, 54.93% came from an urban environment (from the cities of Girona, Figueres and Blanes).

The analysis revealed three overarching themes related to ageing in place: “Meanings and connotations of HA and life quality”, “HA within the community” and “Enablers and barriers to HA in place”. Table 1 shows a comprehensive list of themes and the categories that emerged from the participants’ narratives after analysing the contents of the open-ended questions.

### 3.1. Meanings and Connotations of HA and Life Quality

#### 3.1.1. The Meaning of Ageing

The participants reported that ageing is commonly felt at the onset of health limitations and dependency. Poor motor skills and difficulties such as arthrosis, fatigue, trembling and/or sensory impairments, such as sight and hearing loss, were highlighted as triggers for the perception of ageing. People who suffered or are currently suffering health problems or emotional disorders reported more barriers to achieve a HA. In this sense, “Health” is understood from a holistic point of view. Therefore, both illness and mental health disorders are significant barriers.

*“I believe that HA is to be able to fend for oneself”*

[P1_woman_SG]

“I only feel old, when something falls and I have to bend down to pick it up. Phew…it’s hard!”

[P12_man_SG]

#### 3.1.2. Key Elements for Life Quality in Older Ages

**Figure 2:**
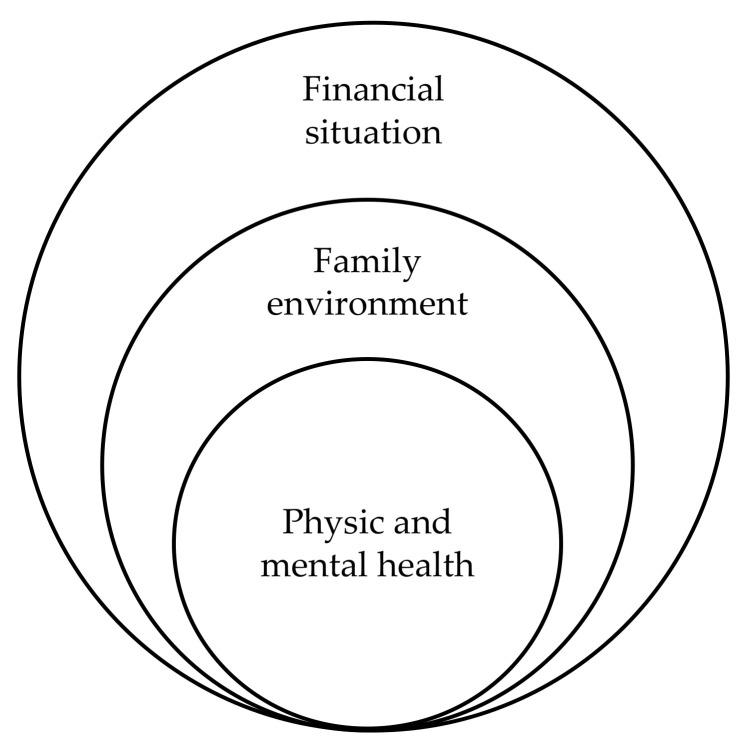
Key elements for life quality in older age.

Life quality was the most remarkable element when thinking about HA. The participants defined “Life Quality” as a group of essential elements required to enjoy life. The most relevant elements stated were: to have sufficiently strong physical and mental health to be able to live without major complications, to have a family around that is doing fine and to have a stable financial situation (Figure 2).

#### 3.1.3. The Ageing Experience

Regarding the HA experience, the participants described ageing as a life stage which is lived differently from previous stages. Participants acknowledged to have learnt to accept the events of life as they come, to be more patient and to live the present day without worrying so much about the future. Accepting the limitations (such as mobility difficulties, physical capacities losses and social changes) and accommodating for what is to come leads to emotional stability. One of the most valuated aspects was the fact of not having to work. There were even some participants who pointed out not having enough time to do all that they wanted to. When analysing the participants’ profiles, those who manifested a more positive attitude were the ones who better accepted the life they were currently enjoying.

*“I do the same. I spend more time, but I do the same.”*

[P6_man_OL]

*“To learn to accept what it comes. To not despair, to resist, and to think another day comes afterwards.”*

[P5_woman_OL]

*“Very much to accept very much the limitations, the limits… Even if you don’t have good health… Calmly...”*

[P3_woman_SG]

*“And to not think much about the future, because you don’t know what will happen.”*

[P1_woman_TA]

*“I have realised that at 80 years old, all that comes is a bonus, so you have to enjoy it to the fullest. Whoever arrives at this stage, should take the most of it.”*

[P12_man_TA]

*“We are doing well; we experienced difficult lives and we have gone through everything. We are even short of time.”*

[P1_man_OL]

Health was considered a key factor to better accept the ageing process. Healthy habits, such as eating and/or physical activity, were acknowledged as a relevant tool for self-care and for enjoying a life in quality during the ageing process. Feeling physically and emotionally strong enabled a better acceptance of the ageing process.

*“Quality of life is also eating well, because when one eats well, the body works better, and one thing helps the other.”*

[P3_woman_OL]

*“A better ageing, to better cope with it. Move, self-care, exercise, watch your diet.”*

[P2_woman_FI]

Emotional and spiritual health were also considered relevant in addition to the physical health. Feeling inner well-being or personal satisfaction was considered a milestone by the participants. Having a clean conscience and being at peace with oneself was regarded as enabling to live the remaining years in a relaxed way. Actually, in view of the results, the perception of ageing was not so much related to the chronological age but rather to the mental age.

*“People have to try to live consciously, that is to say, to acquire a certain peace of mind to cope with the years to come with independence of the health, because health, is difficult to maintain at a certain age and be at a perfect state of health. That is evident, but it’s specially the state of mind of the people that are in peace with themselves, with the way they are living their lives that is important.”*

[P10_man_SG]

*“One thing is to grow up, and another thing is to feel old; I do not feel old. Grown up, I don’t know what to say…”*

[P1_man_OL]

*“Give peace. Feel yourself at peace that is very important.”*

[P7_woman_BL]

The factors that facilitated the acceptance of ageing were found to be the same than the enablers to HA. In this sense, some of the main factors that facilitated a better acceptance of the ageing process was being healthy and/or not having any serious illness, not being in a position of high dependency and having financial stability, whereas the participants who suffered the loss of their partner or close family members, such as offspring, the experience and acceptance of ageing became harder and more painful.

*“When a child gets ill, you get ill too.”*

[P2_woman_SJ]

*“To have quality life means having no problems, to be healthy and to have money.”*

[P1_woman_SJ]

#### 3.1.4. Priorities at Older Age

**Figure 3:**
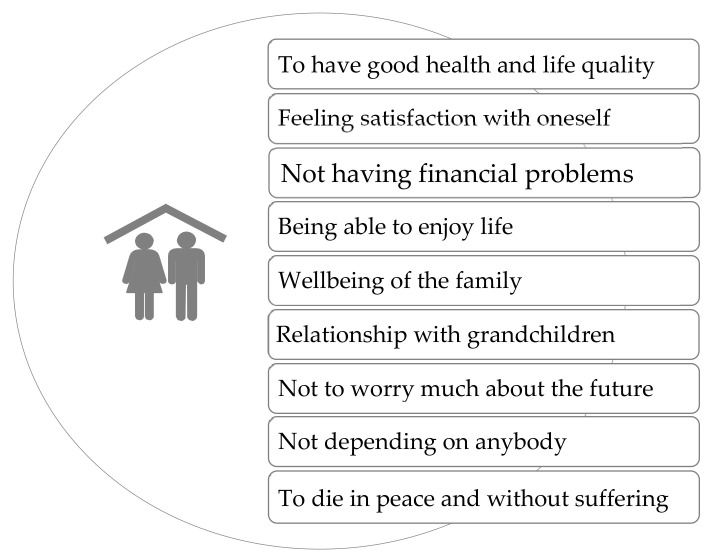
Main priorities in older people’s life.

Figure 3 shows the currently main priorities of the participants.

There were some noticeable differences on the older people’s priorities, depending on the gender of the participants. Women valued more the wellbeing with the family and the relationship with the grandchildren. Such relationship was perceived as very relevant, and with a great feeling of bi-directionality. Older women felt that they received a lot from their grandchildren. Men, on the contrary, preferred to be healthy, to enjoy life quality and to avoid financial problems.

*“It is a satisfaction to feel useful and help the others; and in this case (regarding grandchildren), it is bi-directional.”*

[P12_man_SG]

*“I think the most (important is) peace…and health comes afterwards…like money, money helps.”*

[P9_man_BL]

### 3.2. HA in the Community

Concerning the perception of the older people’s role in the community, the participants manifested that “older people” is a very heterogenic group, with a lot of variety depending on the age, healthy status and attitude towards life.

*“We are older people, but it depends a lot on your age. It is not the same to be 60 than it is to be 80 years old.”*

[P4_woman_OL]

#### 3.2.1. Positive Views

Regarding the role that older people have in the community, positive and negative views were observed. The participants who referred to a positive view stated that older people are admired and respected, especially by their grandchildren. They also accounted that the society is increasingly more concerned about older people, and more activities aimed toward them are organised. Additionally, more intergenerational relationship activities are fostered, to promote a better understanding with younger generations. They highlighted the feeling of being useful and needed, and that they play a role in society, such as taking care of grandchildren or participating in volunteer activities. When analysing the profiles, the participants who lived in smaller villages held a more positive view of the role of the older people in the community. They sensed they belonged to the community and felt well treated, respected and assisted. The participants who considered that their role in society was positive had a higher feeling of belonging in their environment, community and/or family.

*“In our village we have the feeling that we are well treated and respected; the older people get better treatment and attentions; we do not feel neglected.”*

[P3_man_SJ_rural]

*“I think we are taken into account. We try to behave, I do not know, to look for what it is needed, what it is not needed, and I think they recognise it, and we do as well.”*

[P1_man_OL_rural]

*“We live in a village, though it is quite large, it is a village, more or less we do community life, apart from the family, there are the acquaintances… People care, not only friends, but acquaintances.”*

[P12_man_SG_rural]

#### 3.2.2. Negative Views

The analysis of the results showed minor negative views, mostly restricted to a subset of participants, typically men from urban environments, and people who were suffering serious health problems, financial difficulties or lack of family support. Such participants described feeling a lack of respect and low regard towards older people and a sense of feeling invisible to the society.

*“We are ignored, values and the respect towards older people are lost.”*

[P15_man_TA_urban]

*“The society does not take us into account.”*

[P7_woman_BL_urban]

*“I think they see us as useless.”*

[P5_man_BL_urban]

### 3.3. Enablers and Barriers to HA in Place

**Figure 4:**
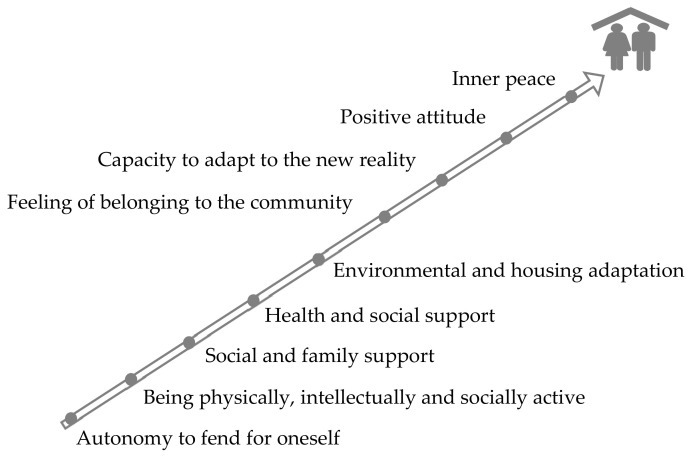
Enablers to HA in place.

**Figure 5:**
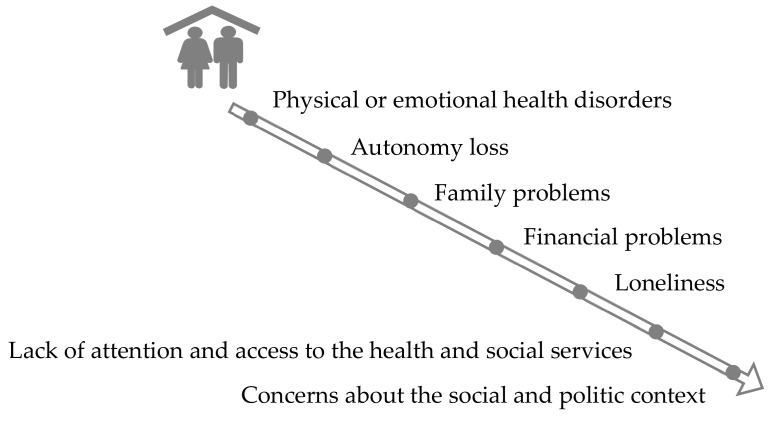
Barriers to HA in place.

The main enablers and barriers to HA in place according to the participants are shown in Figure 4 and Figure 5.

#### 3.3.1. Preserving Function and Autonomy

One of the key elements in the HA in place was the level of autonomy, i.e., the ability to take care of oneself and to live at home up to the limit of feasibility. The home was described as the territory of reference, where they felt free, comfortable and enjoyed their privacy. Dependency is a life condition that the participants would like to be able to avoid. However, they accepted that sooner or later they would have to deal with it. Participants expressed the desire to delay the state of dependency as long as possible, given that it meant becoming a burden, especially for their offspring, whom they considered to be too busy.

*“(…) because at home I am free, comfortable, and I do prefer to be at home with my wife than in any institution.”*

[P9_man_TA]

*“We want to be well as long as possible, to avoid being a burden to our children.”*

[P5_woman_OL]

*“We are getting old, and we will need our children’s help, and I don’t think they can help us…hence, we will have to afford a place in an institution, and even though, it is very difficult to find a place.”*

[P1_woman_SJ]

*“There is an emotional problem, when the older people don’t feel attended…it is not about cooking the meals, doing the washing…As an example: “Daughter! take your mother to walk!” But, as she is always in a hurry, she cannot go. Here is where the emotional problems come…specially for the older people that live at home.”*

[P3_woman_FI]

Additionally, the participants referred that resources and social support are limited, and that private carers are too expensive. Therefore, dependency is a condition they would like to avoid, and it has become one of the main concerns for many of the participants. In some cases, they expressed that the idea of a total dependency condition made them think about death as preferable.

*“Our children will not be able to leave their jobs to take care of us. What can we do? An institution? A hospital? If you have money. But if you don’t, you will remain at home and die on a chair.”*

[P8_woman_OL]

*“What I don’t want is my family to be there…Not me, I prefer to die and that’s all, without suffering, eeh… and not making my children suffer.”*

[P10_man_BL]

*“While I can fend for myself, I prefer to be at home. The day I no longer can, they can let me die if they want it, because to move to whatever place... Firstly, we cannot afford the prices they are charging nowadays. We cannot afford a decent institution.”*

[P12_man_TA]

The institutionalisation was seen as the last option. The perception of the current institutions was very poor, with the feeling that they lack resources, are very expensive (private institutions) or very hard to get admitted (public institutions). The general point of view was that institutions tend to neglect older people. The participants preferred to live at home with the most quality of life for as long as possible, and many of the participants would prefer to die at home, as their parents and grandparents did.

*“In an institution, when you give any problem, they give you double dose and sleep, dazed all the days.”*

[P13_woman_TA]

*“If you have to go to a private, or semi-private institution, it is far too expensive. People cannot afford it… When there is an offspring, it is hard to believe that older people have to go to a nursing home. In the past, everybody lived at home, and now this tradition is lost. It looks as if they are left there, abandoned.”*

[P4_woman_FI]

#### 3.3.2. Financial Stability in Older Age

One of the main concerns of the participants was the prospect of having financial difficulties given the small retirement pensions they received. Participants stated that this financial concern affected their wellbeing, both at an emotional and at a physical level. Additionally, they considered that it is a common concern in the majority of older retired people, as a consequence of having low retirement pensions that make it difficult to make ends meet.

*“Now is when we face more financial problems, even more than in the past; we have three children, we were able to afford their university education… everything was going, we could not save money, but you could make it, but now, with the retirement pension, you cannot make ends meet. This makes me anxious.”*

[P3_woman_BL]

This lack of resources made them feel helpless when thinking about their future. They stated that there are not sufficient resources for dependency, and that they will not be able to afford a private carer or a place in an institution. Therefore, they have to plan ahead their savings, or even use up funds and properties they were planning on leaving as heritage to their families, to pay their future care.

*“The main problem is the financial one; it is a big problem; if you don’t have money, who will take care of you?”*

[P7_woman_SG]

*“One of the things about ageing that I think about is…hell! How are we going to pay!”*

[P1_woman_SJ]

#### 3.3.3. Family and Social Support in Older Age

According to the participants, the family relationships were very important, as they became the key support. Participants mentioned a strong link between the wellbeing and tranquillity of their children and grandchildren and their own wellbeing. The health, employment conditions and financial stability of their offspring were important concerns. They also manifested worries about the employment conditions of their offspring, believing that young people are facing now more difficulties and a more complex job environment than in the past.

*“Being well with the family, with the grandchildren, do all what you can for your children, and not interfere in other people’s life, and keep going forward.”*

[P8_woman_SG]

*“Happily, thanks God, they are healthy and have a job, and that gives me a lot of life.”*

[P6_woman_BL]

*“You are in peace, but the day they say,” Look this company is not doing well”, then not you nor them will be well… It is what most worries us nowadays, that they have a job.”*

[P8_woman_SJ]

The participants highlighted that having a good relationship with their grandchildren made them feel well. Such relationship was stated as crucial and a priority for most of them. They accounted that it was a bi-directional benefit, given that they felt receiving a lot from their grandchildren. The participants manifested to have a lot of contact with their grandchildren, undertaking in most cases a babysitting role, due to the employment conditions of their children. In most cases, this situation was valued positively. The participants accounted that the grandchildren gave them joy, life and felt very much loved by them, in addition to making them feel useful. Nevertheless, there were also some critical opinions that outlined a sense of obligation that they should not have to assume.

*“Grandchildren means 80% (of life). I think this is what makes someone live.”*

[P1_woman_SJ]

*“When the grandchildren come, the pain disappears, and if it does not disappear, I take double dose of medicine.”*

[P4_woman_SJ]

*“An important thing is that we take care of the grandchildren because our children need to work, if not, I don’t know how they would be able to manage it.”*

[P7_woman_SG]

*(Regarding babysitting grandchildren) “The child drains you. It seems that older people have the obligation to take care of everybody.”*

[P4_woman_FI]

Concerning social relationships, the participants manifested that it helped them to be connected with other people, and prevented home isolation and loneliness. Contacting, talking and sharing with other peers, was stated as an enabler to feel they were part of the community, and enhanced their feeling of belonging. To be engaged in the activities of the community centres was outlined as a way to feel part of the community, which, in some cases, was considered as a second family. When analysing the profiles, women and participants from smaller villages demonstrated a more positive attitude toward the familiar and social relationships.

*“What I mean is that I have to defend and fend for myself. I had surgery for thyroid cancer, and lymph nodes. From the third day onward I was at home, alone, nobody helped me. What kept me up was the friendship and company from people of my age. Not the young people.”*

[P8_woman_BL]

*“Going out helps a lot, coming here and talk with someone, then another one. Just with that I feel happier, and it is wonderful. If you have any pain, you come here and the pain goes away because you don’t remember about it anymore.”*

[P2_woman_TA]

*“This helps us, it is like being in a family. Mostly all people that usually gather here look like a family together. We go dancing, because we all know each other, we are friends.”*

[P12_man_TA]

*“Yes, and I like a lot the contact with you all, with people of my age. Because younger people, including my children…I don’t feel completely invisible, just a bit”*

[P6_woman_BL]

The intergenerational relationships promoted in the local social and educational centres were seen as a relevant exchange of knowledge and affection.

Technological devices such as mobile phones and, more precisely, communication applications such as WhatsApp, was remarked to help them to be in constant contact with family and friends. The new technological tools were mentioned as enablers for socialising and making them feel accompanied. The profiles analysis showed that, generally, women used new technology devices more extensively than men. Specifically, women tended to use mobile phones more and men computers.

*“Look, I had one of those old mobiles, and always used to criticise what we were talking about, when my daughters were coming home and always on the mobile, after lunch, and I was suspicious about it, because we could not talk. Finally, they offered me one. The best thing ever. What a company! I am always on the mobile.”*

[P2_woman _BL]

Concerning the experience of loneliness, the participants stated that it was a reality associated with ageing. It was specially highlighted by widowed people and by women. However, participants manifested that it was not the same to be alone than to feel lonely. The importance of learning to live alone was mentioned, together with the concept that women usually live alone better than men. Nevertheless, feeling or being alone was a concern for the majority of the participants, who were afraid of suffering abandonment. This feeling generated fear and anxiety, especially when thinking about the future stages and the uncertainty about who would be taking care of them. It should be outlined that participants from urban environments or larger villages indicated stronger feelings of loneliness than those from rural environments, where there is more proximity and a greater sense of community and belonging. The participants felt more accompanied when they were in the local community centre. The participants proposed measures to address the loneliness to the health and social community services, such as more frequent checks on the people who live alone and to promote activities to enable the contact among older people. They also recommended that people who live alone should not stay at home and should join a group or community.

*“It’s been eight years that my husband died, that I am alone, but I do not feel lonely.”*

[P2_woman_SG]

*“It is very sad. I have a family, but the loneliness itself.”*

[P4_woman_BL]

*“The help button, they tell you, press whenever you need it, but I am not going to be pressing it all day long, and say I feel like crying, they will answer, then cry… That’s why we don’t want to stay at home and feel the world is collapsing. We go out, that’s why we come all in the local centre.”*

[P4_woman_TA]

Another highlighted topic that should be addressed was the loneliness that can be felt when living in an institution.

*“It looks like they are left there, abandoned, because there are some cases that they receive weekly visits, but there are others that do not.”*

[P4_woman_FI]

#### 3.3.4. Proper Care and Access to the Health and Social Services

**Figure 6:**
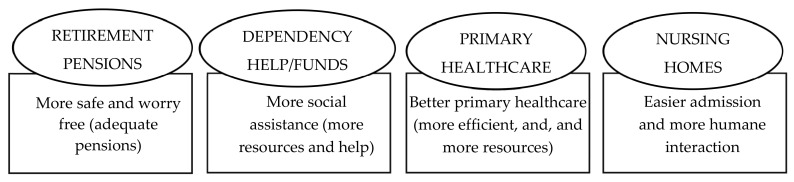
Needs not covered by the health and social public services, according to the participants.

Figure 6 shows the needs not covered by the health and social public services, according to the point of view of the participants, and which would help them in the ageing process.

At a general level, the participants focused on the assessment of the health services. Such assessment was strongly influenced by their personal experience. However, some common items were found: feeling/not feeling attached to the family doctor, frequent changes in the attendant physicians, waiting times and timetable in the health community centre or speciality doctor’s centre and lack of health services during weekends in the smaller villages.

*“I haven’t been lucky. That’s my fourth change in doctor so far.”*

[P10_man_BL]

(Regarding public health) *“In the past, you went to the doctor and they attended you, and you didn’t need to wait as much as you do now…and you feel somehow anxious, because he (the doctor) says you need that but he cannot because of the waiting lists…if you want something quick, you have to go private, and with the retirement pensions.”*

[P15_dona_FI]

Concerning the health services, most of the participants were frequent users of the public healthcare centres. Additionally, many of them had used the health specialist centres due to admissions or surgeries, and globally they felt satisfied about it. Among the positive assessments, it is worth noting the luck to meet an understanding doctor, and the nurses’ role. In both cases, the participants valued especially the empathy towards the patient and the humanisation of care. Other positive assessments included the attention received by the community health centre and the preventive health check-ups. Telemedicine was positive valued when it was a part of the follow up, but not as a substitute of the personal attention and the face-to-face contact. Health assistance at home was not specially highlighted. The participants stated that the acute health disorders usually were solved with the help of the partner or relatives, although they acknowledged the existence of the home assistance service and, in some cases, expressed having used it.

*“I have to score 10 (out of 10) to the nurses, they are vocational.”*

[P6_woman_BL]

*“In the past, people had (high) blood pression, and they did not know it, and they had a stroke. Nowadays we are better controlled in this sense. There is a pill for this and a pill for that.”*

[P8_man_FI]

Concerning the medication, there were both pro-medication and anti-medication opinions among the participants. Pro-medication participants, usually the most affected by serious illnesses, showed gratitude for the help it brings. The anti-medication participants tried to take the minimum medication possible. Generally, all the participants referred to feeling poly-medicated.

*“I try to take the minimum possible.”*

[P4_woman_BL]

*“If they work, I take them; that’s why we live longer.”*

[P3_woman_BL]

*“My aunt, I think she takes ten tablets per day, eh, me, personally, if I need a pain killer or something like that, I don’t take them, I am a bit anti-medication, if, as an example, I have back pain and I can go and lie in bed for five minutes and it disappears.”*

[P2_woman_OL]

An item that participants highlighted regarding the assessment of social services was the role of the older people association centres and the local community centres. Concerning the use of social assistance services, the difficulties getting help in the case of a dependency condition were considered as more problematic. This was a cause of strong concern for them when they considered this need in the future.

*“I think we would have a secure future if the authorities respected the law, I mean, there is the law of dependency that is not followed. There are many people waiting for the help, financial help or carers. And all this is not fulfilled.”*

[P7_man_FI]

*“We are paying so many taxes, all together, every year, and I believe I don’t get the money back in terms of help.”*

[P3_woman_BL]

#### 3.3.5. Activities

The participants manifested that they considered themselves as active and participative people. They were engaged in activities that they liked and helped to feel active and enjoy the life stage they are living. Such activities were seen as a way to help, distract, socialise, feel helpful and fulfil themselves.

*“I feel well, in spite of the current circumstances, because I do things that I like to do…I sing in a chorus, I’m engaged in a havaneres (folk music) group, I go dancing, I am in a musical group in Llagostera, I mean, things that fulfil me.”*

[P2_woman_SG]

*“Given that we have more time, we can do a lot more things than we used to when we had to work…, we can do activities here (local community centre), as an example, or go to the theatre, whatever, above all, the important thing is not to remain at home.”*

[P3_woman_OL]

*“You come here, you are having fun, and you feel like you got rid of your problems, but the problems remain.”*

[P9_man_TA]

The local community centre was reported as being relevant to encouraging and enabling a HA. Many of the participants considered it as their second house. They were very committed in the activities they were engaged there, and that helped them to stimulate their social life. Such centres were defined as places that create community.

The most mentioned activities were: physical activities such as walking, hiking, yoga, tai chi and swimming; intellectual activities such as memory sessions and language and informatics courses; hobbies such as singing, dancing, cooking, horticulture, crochet, mushroom picking, fishing and travelling; and finally volunteer activities.

*“It’s great, because the activities are open to people of all kinds, whoever can do more, or less, because even those who cannot walk, can engage in crochet, whoever likes studying, there are Catalan, English, whatever classes, there is yoga, gymnastics, of course, people than can do that, they do it, and who cannot, does less.”*

[P1_woman_SG]

*“With the vegetable garden, there is a huge satisfaction when you see everything grow… and you can give, I always plant more to give to friends and neighbours, and it is a great satisfaction.”*

[P1_man_OL]

#### 3.3.6. Environmental and Housing Adaption for the Ageing Process

The participants evidenced the need that both the houses and the urban environments should be better adapted to the older people. The most frequent adaptations conducted at home were to prevent falls. Some participants accounted to having suffered falls due to loss of motor skills and balance. Women reported more episodes of falling than men, due to arthrosis or osteoporosis. Consequently, the most frequent adaptation was the removal of the bath and its replacement with a shower to facilitate the access and the addition of handrails to prevent slipping. In some cases, stumble problems due to the height of sidewalks were reported, as well as slipping in the streets on rainy days.

*“Falls are terrible... I take my cane; some people don’t because they are ashamed of it.”*

[P3_woman_OL]

*“When we moved, the first think I said: a shower.”*

[P6_woman_SJ]

Regarding the barriers to the mobility and access to their buildings or houses, one of the issues that was highlighted was the lack of a lift in the buildings, and consequently difficulty of using the stairs. Such concern was more common in the participants who lived in old or peripheral neighbourhoods of urban environments.

*“I live in the first floor, but there is no lift, and the stairs are hard for me.”*

[P2_woman_TA]

*“I broke my leg and I had no choice but to go up the stairs. You don’t know what I have been through with the stairs. I would raise a foot, and then the other I couldn’t raise it anymore, dragging, dragging, and raise the foot another bit again.”*

[P10_man_TA]

*“It’s been two years that my wife cannot go up the stairs to the bedrooms anymore. We had to put in the dining room, we had to split it and put a room there. And there we have dining room, bedroom, bath and kitchen, in one piece, like this. All due to the lack of resources to go upstairs. And when it is needed, I go up, but she cannot.”*

[P9_man_TA]

The location of the housing was stated as a relevant enabling factor. A location near the neighbourhood facilities, with easy access, is crucial. Regarding the neighbourhood, participants highlighted positive aspects, such as having good neighbours, having facilities nearby and a peaceful environment. Negative aspects of the neighbourhoods had to do mainly with being dirty or poorly maintained. The more negative views were expressed by participants who lived in urban environments of larger cities.

*“I would hold (the house) and would put it here, in the centre of the village, because I have to go uphill and it is getting more difficult every day.”*

[P3_woman_BL]

*“The only thing is the cleanliness, we feel abandoned… As if we were not paying our taxes.”*

[P12_man_TA]

## 4. Discussion

Regarding the meaning and connotation of ageing, the participants generally expressed that, in their opinion, ageing starts when the health limitations and dependency begin. HA is equated to life quality, which is relevantly determined by emotional, social and financial factors. Therefore, HA is mainly conditioned by the physical and emotional health, as well as by social aspects, encompassing a multidimensional perspective of the concept of health [45]. It is worth mentioning that, in 2018, the number of healthy life years at birth was estimated at 64.2 years for women and 63.7 years for men [46], while the life expectancy at birth in Europe for the same year was 81.0 years (78.3 years for men and 83.6 years for women) [47]. Therefore, it can we concluded that most older people live their last 15–20 years suffering from health problems and/or disability. In this line, the outcomes of the present study highlight the reality of people between 62 and 92 years old who, despite the physical health problems typically associated with age, live their current life stage adapting themselves to the ageing limitations and accepting what the day-to-day life brings [25]. This observation indicates that, together with the healthy habits, emotional health and interior peace are very relevant. Although a number of the participants stated that ageing affects a wide range of different age groups, in the explanations of the participants, the most relevant differences were found in aspects such as the health state at present, the attitude towards life and their own perception of being and feeling old. All the participants agreed to identify ageing in place as a key element of their quality of life [23,48]. Health is a crucial factor to enable the possibility to keep living at home, but not the only one. Other factors considered to be critical were the financial status and the family environment, in agreement with the study conducted by Wilhelmson et al. [48]. The participants’ priorities in ageing are well aligned with the enablers and barriers to HA in place described. It should be outlined that women stressed the relationship with their grandchildren, and men the financial aspects. These two factors are in agreement with two of the eight key elements for ageing in place described by Kennedy et al. [25].

Regarding the role of older people in the society, it is worth highlighting that participants who referred a more positive view were the ones who had a stronger feeling of belonging, either because they were from a small village or rural area, or because they had a higher level of social engagement. In this sense, Wiles et al. [26] stated that ageing in place is related to a sense of identity both through independence and autonomy and through relationships and solidarity roles in the places people live. Conversely, the more negative opinions came from people who lived in urban environments or had experienced difficulties during their ageing process.

The participants who did not have a high level of dependency at the moment of the study expressed the wish to live in place as long as possible. This fact agrees with what other authors highlighted about older people with low dependency levels and their preferences for ageing in place [10,11,12,13,14,17]. Institutionalisation is a traumatic concern they prefer to delay as much as possible and considered as the last option in the event of dependency and physical impairment [17]. The high expenses of institutionalisation make it difficult for them to access this service, which also represents a future concern that was frequently commented. In this sense, both Mascarilla and Cresí [17] and Kendig et al. [18] linked the fact of having less affluence with being less likely to opt for institutional care. The financial status of older people can also be a relevant barrier to ageing in place in healthy and comfortable conditions. Many participants referred to having low retirement pensions that caused them concern regarding the possibility to save money for a private carer or a place in an institution in the future. Additionally, the perception of the service provided by the nursing homes is mostly negative, and, in some cases, participants stated to prefer dying than leaving their home. Such broadly negative perception about institutionalisation supports the need to implement the Fourth Strategic Objective—“developing sustainable an equitable systems for providing long-term care”—proposed in the Global Strategy and Action Plan on Ageing and Health [7].

The support of the social and familiar environment is an equally relevant element that can influence the ageing in place, in agreement with Zueras and Ajenjo [22]. The family is seen as a generator of willingness to live and an enabler to ageing with quality. However, it is also referred to as a barrier in the cases where problems and concerns about family wellbeing appear. Older people wish for family wellbeing, and therefore try to help the family members within their capabilities. The relationship with their grandchildren is mentioned very often. According to the reports of the Institute of Elderly Social Services (IMSERSO), most young people aged between 18 and 24 years (83.4%) are currently in contact with their grandparents [49]. The participants expressed different points of view regarding their grandchildren. The majority considered them as a source of happiness, while in smaller groups of participants identified the care of the grandchildren as a burden. Generally, women accepted better the role of carers. Although older people had the perception that they were engaged in the use of new technologies (women through the use of mobile phones to connect to social network applications and men through the use of computers for conducting Internet searches), the reality, according to the reports of the IMSERSO, is that only 11.9% of young people (18–24 years old) connect with their grandparents through WhatsApp or other social networks [49].

Living in place is an important factor of connexion [23] and social inclusion [26], especially if people go out and engage with entities of social support, such as in the case of the present study. The feeling of belonging to the house and to the community is very relevant. This feeling is mostly manifested through the active engagement to the local social centres, where the participants create a social and supportive environment. There is a general agreement that participating in the activities offered by the local social centres is a determinant factor to better accept ageing and feel less lonely. Being less engaged in social activities increases the likelihood of older people moving away from their houses [24]. As such, participation in social activities is a preventing measure against institutionalisation [50]. The participants of the present study were engaged to different social activities that increased their feelings of belonging to the community, peer relationships and enabled them to better fight loneliness. In this sense, loneliness was stated as a reason for concern, due to their knowledge of other people suffering from it in their community. Additionally, participating in activities in the social centre enables to keep them physically and mentally active. Therefore, it is clear the role of local social centres as a very valuable health asset for older people.

Furthermore, in line with the third strategic objective of the Global Strategy and Action Plan on Ageing and Health [7], it was observed that there is the need of integrated, responsive and accessible primary health and community services to support a successful ageing-in-place [25]. The participants had a positive assessment of the attention received from health professionals, but a negative opinion regarding the lack of efficiency and resources. The main complaint was focused in the social resources: pensions, dependency help funds and accessibility to nursing homes. They believed that if the pensions and dependency help funds were adequate, they would be able to remain at home and avoid institutionalisation. Such outcome goes in line with other studies [51] that stated that socially disadvantaged elderly people perceive greater barriers to accessing healthcare services than those who are better off. To promote ageing in place, more attention should be given to the impact of the stress that carers and families suffer. Consequently, better actions could be envisioned to give them a better support [23]. It is worth mentioning that none of the participants discussed intermediate options between living at home and living in an institution. The possibility to have a private carer at home, as an intermediate option, was seldom mentioned and was described as totally unaffordable.

According to the participants, their house is the space of intimacy, where they feel free and comfortable. However, concerning the home conditions and facilities, some participants outlined the lack of a lift in their building as a relevant barrier. This was mentioned as a limitation that they could cope with, but with effort and difficulties while the level of dependency was still low. In this line, the study by Safran-Norton [52] concluded that living in a multifamily building without a lift was a predictive factor to the institutionalisation in older people living as a couple, whereas living in a uni-familiar house with small interior adaptations was a predictive factor for remaining at home for single people. Most of the participants in this study conducted small renovation work at home, such as removing a bathtub for a shower and installing handrails in the bathroom. In a longitudinal study conducted in Australia, it was concluded that having adaptations at home was linked to ageing in place [18]. Notwithstanding, another study suggested that people may delay having adaptations, because of perceived stigmatising associations with decline and vulnerability [53]. Fear of stigmatisation related to adaptation work at home was not mentioned in the FG of the present study. On the contrary, it was well accepted except for being costly, or in the cases that the participants were not the house owners. Concerning the living environment, participants expressed the relevance of having easy access to general facilities, accessible and safe sidewalks, and cleanliness in the urban areas.

The housing situation of older people in Spain is characterised by social inequality, with situations of extreme vulnerability as a result of insufficient policies, public cutbacks and changes in the familiar solidarity. In this sense, the social welfare system is not addressing the housing needs of older people, therefore hindering the ageing in place and the integration of older people within their community [9]. If governments were to embrace the goal of ageing in place, they would have to promote actions to improve the adequate environment conditions, both at home and in the neighbourhoods [24,54]. This is especially important if we take into account the predicted future tendency towards living at home, given development of technology and human resources in the housing environment [55].

As a summary, this study shows how older people describe the main determinants in the ageing in place, which are related to those described in Rolls et al. [21]: habitat and safe environment, sufficient income to sustain life, support from family and friends and access to primary healthcare. Actually, the WHO definition of HA mentions the link between the functional ability determined by the intrinsic capacity, the environmental factors, which include housing, social facilities and healthcare, relationships with friends and family and the interaction of this two elements [5]. A better understanding of the experience of ageing within the community will enable a more focused approach to the challenge that societal ageing represents. This knowledge would potentially improve policies and health promotion actions in elderly, which could contribute to promote older people to be more active, autonomous and integrated in their living environment.

The main limitation of the present study is that it explored the reality of HA in place from the perspective of older people who are actually integrated in their community, through their engagement in activities in the local community centres. As such, this only reflects a partial, although important, view of a larger reality. Another limitation comes from the fact that no data was collected on whether the participants lived alone or shared their home with other people. A better understanding of the impact of living alone would add value to the findings. Therefore, there is the need to proceed with research on the reality of older people who live at home but are not integrated in their community, either because they have a high level of dependency, or because they suffer from loneliness. Other important research lines that stem from the work of this study are ageing in place in the socially disadvantaged elderly people, and the inequities that are present within the older people. Further longitudinal studies, such as The Survey of Health, Ageing and Retirement in Europe (SHARE), would provide a deeper knowledge of which enablers and barriers to HA in place are more relevant to prevent or delay the institutionalisation of older people and contribute, therefore, to the sustainable development of the society.

## 5. Conclusions

The different ways older people experience their ageing process depends on the life stage they are living at present, their health conditions, on the attitude towards and the acceptation of such process. The perception of ageing is linked to the psychological and emotional status more than to the chronological age itself. Ageing in place was seen as an important goal, for which good health and life quality are fundamental aspects to achieve it.

The enablers to HA in place are: autonomy to fend for oneself; physical, cognitive and social activity; social and family support; adequate care and access to the health and social services; feeling of belonging; capacity to adapt to the new reality; house and environmental adaptations; and inner peace. Identified barriers to HA in place were the poor physical or emotional health, loss of autonomy, family problems, loneliness, financial difficulties, lack of care or access to the health and social services and concern about the social and political context.

The promotion of older people’s autonomy and wellbeing, together with the creation of an active network of health and social services, may improve the prospects for older people to live their ageing process at home and avoid or delay the institutionalisation.

## Figures and Tables

**Table 1:** Comprehensive list of themes and categories identified after thematic analysis.

Elderly’s Perceptions about Ageing in Place	Theme 1.	Theme 2.	Theme 3.
	Meanings and Connotations of HA and life quality	HA within the Community	Enablers and Barriers to HA in Place
Categories	Meaning of ageing	Positive views	Preserving function and autonomy
	Key elements for life quality in older ages	Negative views	Financial stability in older age
	Accepting the ageing process		Family and social support in older age
	Priorities at older age		Proper care and access to health and social services
			Activities
			Environmental and housing adaptation to the ageing process

* Includes older men and women of different age groups, over 65 years old.

**Figure A1:**
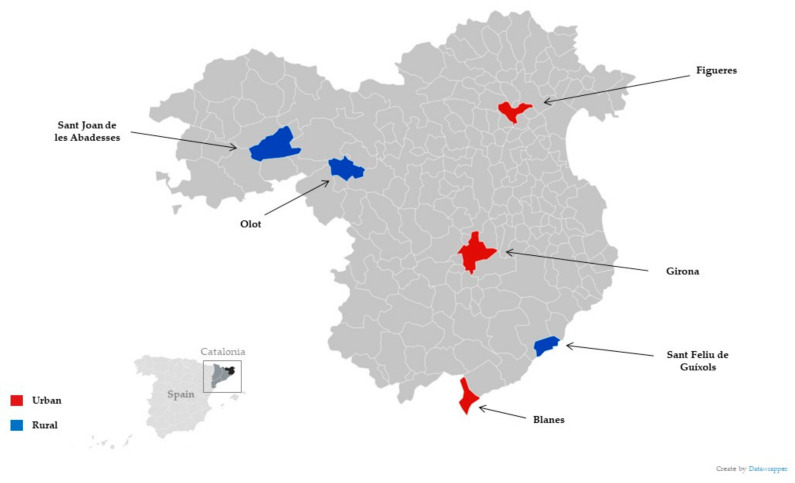
Health Region of Girona in Catalonia (Spain), with the municipalities of the study area.

## Appendix B

**Table A1:** Settings of the group sessions described according to the geographic location and the rural or urban environment *.

Area	Counties (Comarques)	Cities/Villages	Entities
Urban	Gironès	FG 1: Taialà (TA)	Casal de la Gent Gran de Taialà (senior activity centre)
	Alt Empordà	FG 2: Figueres (FI)	Casal de la gent gran de Figueres (senior activity centre)
	La Selva	FG 3: Blanes (BL)	Casal de la Gent Gran Benet Ribas de Blanes (senior activity centre)
Rural	Baix Empordà	FG 4: Sant Feliu de Guíxols (SF)	Casal Gran Tueda Apite (community activity centre)
	Garrotxa	FG 5: Olot (OL)	Associació Gent Gran d’Olot (old people’s association centre)
	Ripollès	FG 6: Sant Joan de les Abadesses (SJ)	Associació Jubilats St. Joan de les Abadesses—Casal Jaume Nunó (retirement activity centre)

* Includes older men and women of different age groups, over 65 years old.
